# Source-Free Class-Balanced Pseudo-Label Adaptation for Large-Scale Cross-Day WiFi Transmitter Identification

**DOI:** 10.3390/s26185975

**Published:** 2026-09-21

**Authors:** Yuheng Yang, Haiying Zhang, Xiaoya Wang, Mingdi Li, Jiafeng Li

**Affiliations:** The 54th Research Institute of China Electronics Technology Group Corporation, Shijiazhuang 050081, China

**Keywords:** RF fingerprinting, WiFi transmitter identification, cross-day identification, source-free domain adaptation, pseudo-labeling, class balancing

## Abstract

Radio-frequency (RF) fingerprint distributions vary across collection dates, and changes in propagation conditions and hardware states can degrade transmitter-identification performance. To address this issue, we propose Source-Free Class-Balanced Pseudo-Label Adaptation (SF-CBPA) for cross-day WiFi transmitter identification. SF-CBPA requires only an unlabeled target-day dataset and a pretrained source model. It recalibrates batch-normalization statistics, freezes the source classifier, and uses Sinkhorn-balanced pseudo-labeling with within-class confidence selection; an exponential-moving-average teacher and information maximization provide auxiliary stabilization. Experiments were carried out using the public WiSig-ManyTx subset with 140 transmitters and one fixed receiver. The first three collection days trained the source model, while Day 4 provided 25 unlabeled adaptation samples and 25 independent test samples per class. Across three random seeds, SF-CBPA achieved 63.80 ± 0.90% accuracy and 0.6330 ± 0.0121 Macro-F1. A protocol-aligned SHOT implementation achieved 61.72 ± 1.95% accuracy and 0.5963 ± 0.0285 Macro-F1, while source-only and TENT-style achieved 57.93 ± 0.58% and 58.58 ± 0.43% accuracy, respectively. Direct analysis shows that Sinkhorn reduced the hard pseudo-label count range from 1–54 to 13–35 and the class-count standard deviation from 11.75 to 3.42. Component-isolated ablation identifies Sinkhorn balancing as the principal contributor under seed 123; the independent benefits of EMA and the auxiliary information-maximization term are small or inconsistent. These conclusions are restricted to the balanced, closed-set, fixed-receiver protocol evaluated here.

## 1. Introduction

Radio-frequency fingerprint identification (RFFI) exploits device-specific imperfections in a transmitter’s radio-frequency front end that are difficult to reproduce exactly, thereby distinguishing wireless devices of the same nominal model from their received signals [1,2]. By providing complementary physical-layer evidence, RFFI has potential applications in Internet-of-Things access authentication, rogue-device detection, and wireless network security [3,4]. Unlike software-defined identifiers such as media access control addresses, RF fingerprints originate from the combined nonidealities of oscillators, power amplifiers, digital-to-analog conversion chains, in-phase/quadrature branches, and modulation processes. Deep neural networks can learn discriminative representations directly from raw or lightly preprocessed I/Q sequences and have demonstrated strong transmitter-identification performance under controlled in-domain conditions [5,6].

However, high accuracy obtained from random same-day data splits does not necessarily imply long-term reliability in practical deployments. Receiver-chain states, propagation environments, device temperature, carrier-frequency offset, phase noise, and automatic gain-control settings may vary across collection dates. Consequently, date-related nuisance factors and intrinsic device-specific characteristics coexist in the observed signal [7,8]. When the training and test signals are collected on different days, a deep classifier may rely partly on incidental statistics associated with the source dates rather than exclusively on stable transmitter characteristics. This mismatch produces cross-day distribution shift and can substantially degrade identification accuracy. In large-device networks, repeatedly collecting and labeling new signals from every registered transmitter is costly and difficult to sustain. Moreover, privacy, communication-bandwidth, intellectual-property, or data-retention restrictions may prevent the original source-domain I/Q samples from being stored or transmitted to the deployment side.

Existing studies have addressed RF-domain distribution shift through data augmentation, feature alignment, pseudo-labeling, continual learning, and test-time adaptation. Conventional unsupervised domain adaptation generally assumes simultaneous access to labeled source data and unlabeled target data during adaptation [9,10]. Source-free domain adaptation instead retains only a pretrained source model and unlabeled target-domain samples [11]. TENT minimizes target prediction entropy and updates the affine parameters of batch-normalization layers [12], whereas SHOT freezes the source classifier and adapts the target feature extractor through information maximization and pseudo-label self-training [11]. Because SHOT closely matches our information-access constraint, we include it as the strongest general source-free comparison. Our method addresses an additional failure mode in large-class cross-day RFFI; highly concentrated pseudo-label counts can make easy classes dominate self-training while difficult classes receive weak supervision.

To investigate this problem under a controlled cross-day setting, we constructed a closed-set recognition protocol from the public WiSig–ManyTx subset. WiSig contains signals collected from multiple transmitters, receivers, and dates, making it suitable for studying receiver- and date-related variations in RF fingerprints [7]. Receiver 18-2 was selected solely according to four-day data completeness, and 140 transmitters for which 50 samples were available on each of the four collection dates were retained. The receiver was fixed throughout the experiment, so that the principal evaluation factor is temporal distribution shift rather than the combined effect of receiver and date variation. Data from the first three collection days constitute the labeled source domain. For each transmitter, the 50 signals collected on Day 4 were divided into 25 unlabeled adaptation samples and 25 independent test samples. The two subsets had nonoverlapping sample indices, the adaptation files contained no label field, and the independent test set was accessed only after target adaptation had been completed.

Based on this protocol, we propose Source-Free Class-Balanced Pseudo-Label Adaptation (SF-CBPA) for large-scale cross-day WiFi transmitter identification. During adaptation, SF-CBPA requires only a pretrained source model and unlabeled target-day signals. It first recalibrates batch-normalization statistics using the target adaptation set and freezes the source classifier to preserve the learned class hypotheses. An exponential-moving-average teacher is employed to produce temporally stable target predictions. Sinkhorn-based balanced assignment then constrains the global pseudo-label distribution and prevents target samples from collapsing into a few dominant classes. Because balanced assignment alone cannot guarantee pseudo-label correctness, confidence-based selection is subsequently performed within each predicted class, retaining relatively reliable samples while maintaining coverage of difficult classes. The target feature extractor is optimized using pseudo-label cross-entropy together with an auxiliary information-maximization objective. The methodological contribution is a problem-oriented integration of AdaBN, Sinkhorn normalization, exponential moving averaging, and information maximization, with class-coverage control separated from within-class reliability filtering and systematically validated under a large-class source-free cross-day RFFI protocol.

The main contributions of this study are summarized as follows. First, we formulate a strictly isolated source-free cross-day WiFi transmitter-identification protocol involving 140 transmitters, one fixed receiver, source training on the first three collection days, and separate unlabeled adaptation and independent test subsets on Day 4. Second, we develop SF-CBPA to combine target-domain batch-normalization calibration, a frozen source classifier, an EMA teacher, Sinkhorn class-marginal control, within-class high-confidence selection, and auxiliary information maximization. Third, we evaluate Source-only, AdaBN, TENT-style, and SHOT over three random seeds and add direct pseudo-label-distribution analysis, component-isolated ablations, ρ/$\lambda_{\mathrm{IM}}$ sensitivity, paired statistical tests, sample-size sensitivity, and computational analysis. The new evidence distinguishes the dominant contribution of Sinkhorn balancing from the smaller or seed-dependent effects of the auxiliary components.

Across three random seeds, SF-CBPA achieved 63.80% mean accuracy and 0.6330 Macro-F1 on the independent Day 4 test set. It improved mean accuracy over Source-only, TENT-style, and SHOT by 5.87, 5.22, and 2.08 percentage points, respectively. SHOT itself improved over the lighter Source-only and TENT-style baselines, confirming that source-hypothesis transfer is a strong comparison. The direct distribution and component-isolated analyses show that Sinkhorn markedly reduced pseudo-label concentration and accounts for the clearest single-component performance change. EMA did not improve the seed-123 result when isolated, so it is retained as a temporal-smoothing design choice rather than claimed as an independently validated source of gain.

The remainder of this paper is organized as follows. Section 2 reviews cross-day RFFI, source-free adaptation, class-balanced pseudo-labeling, and the task setting. Section 3 presents SF-CBPA, leakage prevention, comparison methods, and implementation details. Section 4 reports overall performance, statistical tests, direct pseudo-label analysis, component ablations, sensitivity results, class-level behavior, and complexity. Section 5 discusses the mechanism, deployment significance, generalizability, and limitations. Section 6 concludes this paper.

## 2. Related Work and Task Setting

### 2.1. Cross-Day Stability and Robust Identification in RFFI

Early RF fingerprinting methods commonly relied on transient features, steady-state spectral features, modulation errors, and handcrafted statistics [1,2], whereas deep networks can learn hierarchical representations directly from I/Q sequences [5,6]. Riyaz et al. used convolutional networks to identify devices from raw wireless signals [5], and ORACLE further investigated the ability of convolutional networks to model differences among wireless devices [6]. These studies advanced end-to-end RFFI, but many experiments used random splits within the same collection session, where domain shift was relatively limited. Recent reviews and model studies have extended the focus to multiple protocols, including LoRa and WiFi [3,4], and have improved representation under complex conditions through multi-feature fusion [13], residual architectures [14], and channel equalization [15].

The WiSig dataset contains 174 commercial WiFi transmitters, 41 USRP receivers, and four collections completed within one month, totaling approximately ten million packets; it was designed to support large-scale research under receiver and channel variation [7]. Its compact ManyTx subset contains 150 transmitters, 18 receivers, four dates, and at most 50 signals for each combination. The original WiSig study showed that receiver and date variation reduce identification performance [7], and subsequent long-term evaluation also demonstrated observable temporal variation in RF fingerprints [8]. Cross-day protocols are therefore closer to practical deployment than same-day random splits but are substantially more difficult. Meanwhile, federated learning [16], channel-robust features [17], noise-robust open-set identification [18], multipath robust and spectral-regrowth features [19,20], prototype-based open-set detection [21], and channel-estimation-based identification [22] have become important deployment-oriented research directions.

Recent studies of cross-day specific-emitter identification have begun to use unsupervised domain adaptation. For example, Wu et al. combined adversarial domain alignment, confidence-based pseudo-labeling, and cross-domain contrastive regularization to evaluate multiple date-transfer tasks on WiSig–ManySig and LoRa data [9]. This line of work demonstrates the value of domain adaptation for cross-day identification. The present study complements source-accessible UDA by considering a source-free setting in which source I/Q samples are unavailable after training and by evaluating the larger-class ManyTx subset. Because the data scale and information-access conditions differ, the discussion focuses on methodological positioning rather than direct numerical comparison. Studies published in 2024–2025 have further advanced RFFI robustness through cross-receiver domain adaptation [23], cross-time continual learning [24], few-shot feature separation [25], source-free transfer [26], contrastive learning [27], receiver-agnostic identification [28,29], dynamic distribution alignment [30], and feature disentanglement [31].

Taken together, research over the past two years shows that RFFI robustness is shifting from in-domain accuracy comparisons toward deployment constraints involving time variation, receiver variation, low SNR, few-shot data, and open-set recognition. Existing methods improve generalization through channel suppression, feature disentanglement, domain alignment, contrastive learning, and generative denoising, but most still require source data, target labels, or additional receiver data. The fixed-receiver, cross-date, source-inaccessible setting adopted here is intended to incorporate both temporal drift and information-access constraints while avoiding confounding cross-receiver and cross-day differences.

### 2.2. Source-Free Adaptation and Class-Balanced Pseudo-Labels

Domain-adversarial neural networks learn domain-invariant representations through gradient reversal and are representative UDA methods [10]; their adaptation stages require simultaneous access to source and target domains. AdaBN replaces source-domain batch-normalization statistics with target-domain statistics, providing distribution calibration without parameter updates [32]. TENT minimizes prediction entropy at test time and updates only batch-normalization affine parameters [12]. These methods have low deployment costs, but under severe cross-day shift with many classes, first- and second-order statistics or entropy minimization alone may not correct systematic class bias. Recent cross-receiver studies likewise indicate that domain alignment [23,30], dynamic pseudo-labeling [29], and feature transfer without source-data access [26,31] are important for deployment generalization.

Source hypothesis transfer (SHOT) introduced source-free unsupervised domain adaptation by freezing the source classifier and adapting the target feature extractor through information maximization and pseudo-label-based self-training [11]. It is therefore the closest general source-free reference to our setting. SF-CBPA preserves the source-hypothesis principle but targets concentrated pseudo-label counts in large-class cross-day RFFI through EMA predictions, Sinkhorn-constrained soft assignments, and within-class confidence ranking. We now include a protocol-aligned SHOT implementation under the same ResNet1D backbone, Day 4 split, adaptation budget, and three source checkpoints, enabling a direct numerical comparison rather than only a methodological discussion.

This study combines class-balanced pseudo-labeling with teacher–student updating. Pseudo-labels depend on the model’s current predictions; if the initial target predictions are class-biased, a simple global confidence threshold repeatedly selects only a few high-confidence classes. Sinkhorn–Knopp normalization can compute approximately balanced soft assignments under prescribed marginal constraints and has been used for unsupervised clustering assignments [33]. We use this optimization tool to constrain the class marginal of a target batch or the complete target set to be approximately uniform and then perform high-confidence selection within each predicted class. In this study, Sinkhorn normalization serves as an anti-collapse mechanism for pseudo-labels in large-class cross-day RFFI and is validated under a strict source-free protocol. In unsupervised or weakly supervised SEI, contrastive clustering, group-label construction, and noise-robust generative models similarly emphasize the need for stable supervision and prevention of error accumulation [16,34,35].

A teacher–student framework forms smoother teacher predictions through an EMA of student weights [36]. Information maximization reduces conditional entropy while encouraging prediction diversity [11]. In SF-CBPA, these mechanisms are auxiliary to balanced pseudo-label construction. The component-isolated seed-123 ablation shows that removing Sinkhorn reduced accuracy by 2.45 percentage points, whereas removing within-class selection reduced it by 0.23 points and removing information maximization by 0.03 points. Removing EMA increased accuracy by 0.49 points for this seed, indicating that EMA supplies temporal smoothing but it did not provide a consistent independent accuracy gain in the present experiment.

From an optimization perspective, class collapse is not merely a lack of confidence; it results from the mutual reinforcement of initial target-domain bias, sample-selection bias, and gradient-update bias. A global threshold preferentially retains easy classes, and ordinary self-training then embeds this imbalance into the feature space. Increasing prediction sharpness alone therefore cannot ensure effective supervision for every class. We first use Sinkhorn to constrain global class coverage and then select relatively reliable samples within each class, so that coverage and reliability are controlled by separate steps. This mechanism also provides a testable explanation for why class balancing is the principal source of improvement in the subsequent ablation study.

### 2.3. Research Positioning and Task Setting

Table 1 compares representative studies from the perspectives of task, adaptation information, and validation scale. The comparison positions this work within the existing literature while accounting for differences among datasets and protocols. The original WiSig study and the long-term stability study revealed receiver, channel, and temporal variation [7,8]. TENT and SHOT provide general test-time/source-free adaptation paradigms [11,12], whereas Wu et al. developed a source-accessible UDA framework for cross-day SEI [9]. This study lies at their intersection; RFFI is the task, source-free access is the information constraint, ManyTx-140 is the large-class validation scenario, and class collapse in balanced pseudo-labeling is the specific problem.

Methodologically, this work provides a structured solution to a specific engineering problem by integrating source-hypothesis preservation, target-statistics calibration, class-marginal control, and within-class reliability filtering. Table 1 is a conceptual positioning table rather than a numerical cross-study ranking because the listed studies use different datasets, access assumptions, and evaluation protocols. Numerical conclusions in this article are therefore restricted to methods evaluated with the same ResNet1D backbone and ManyTx-140 split.

In the task setting, the data protocol itself is treated as part of the method validation. The fixed receiver reduces receive-chain differences, the first three dates cover temporal variation within the source domain, and Day 4 is divided equally at the device level into an unlabeled adaptation set and an independent test set. This design gives the class-balance prior a clear empirical basis and allows subsequent statistical tests to compare paired predictions on the same 3500 test samples. Thus, Section 2 not only reviews related work but also explains the choice of the ManyTx-140, closed-set, batch source-free adaptation setting.

Let the labeled source domain consist of the first three collection days, denoted by $D^{s}=\{(x_{i}^{s},y_{i}^{s})\}$, where $x_{i}^{s}\in R^{2\times 256}$ is a complex-baseband I/Q sequence and $y_{i}^{s}\in \{1,\ldots ,C\}$ is the transmitter class, with *C* = 140. The source model is written as $f_{s}=g_{s}\circ h_{s}$, where $h_{s}$ is the feature extractor and $g_{s}$ is the linear classifier. The target date is divided into an unlabeled adaptation set $D^{\mathrm{ta}}=\{x_{j}^{\mathrm{ta}}\}$ and an independent test set $D^{\mathrm{te}}=\{(x_{k}^{\mathrm{te}},y_{k}^{\mathrm{te}})\}$. During adaptation, the algorithm may access only $f_{s}$ and $D^{\mathrm{ta}}$, but not $D^{s}$, $y_{j}^{\mathrm{ta}}$, or $D^{\mathrm{te}}$; $D^{\mathrm{te}}$ is used only once for final evaluation after adaptation. We assume a closed-set recognition scenario, meaning that the source and target domains share the same class set.

To avoid ambiguity, adaptation may access only the pretrained source model and the unlabeled target adaptation set. The independent target test set remains inaccessible until the final evaluation.

This task is neither conventional supervised fine-tuning nor UDA with simultaneous access to source and target domains. The adaptation algorithm receives an unlabeled target sample set that has undergone domain shift; its only stable semantic anchor is the 140 output directions of the source classifier. We therefore freeze the classifier and adapt the feature extractor so that target features move back toward the original class hypotheses. The class-balancing constraint uses the equal per-class sample count in the experimental protocol as a prior and prevents unlabeled optimization from destroying class coverage. The principal notation and assumptions are summarized in Table 2.

WiSig collected 2.4 GHz WiFi signals on the Orbit testbed; it provides raw data, fully processed data, and several compact subsets [7]. Each identified signal contains 256 complex-baseband samples; both unequalized preambles and equalized signals are available. We use the compact ManyTx subset, whose original dictionary contains fields including tx_list, rx_list, capture_date_list, equalized_list, max_sig, and data. ManyTx includes 150 transmitters, 18 receivers, and four collection dates (1 March 2021, 8 March 2021, 15 March 2021, and 23 March 2021), with at most 50 signals for each transmitter–receiver–date–version combination.

**Figure 1:**
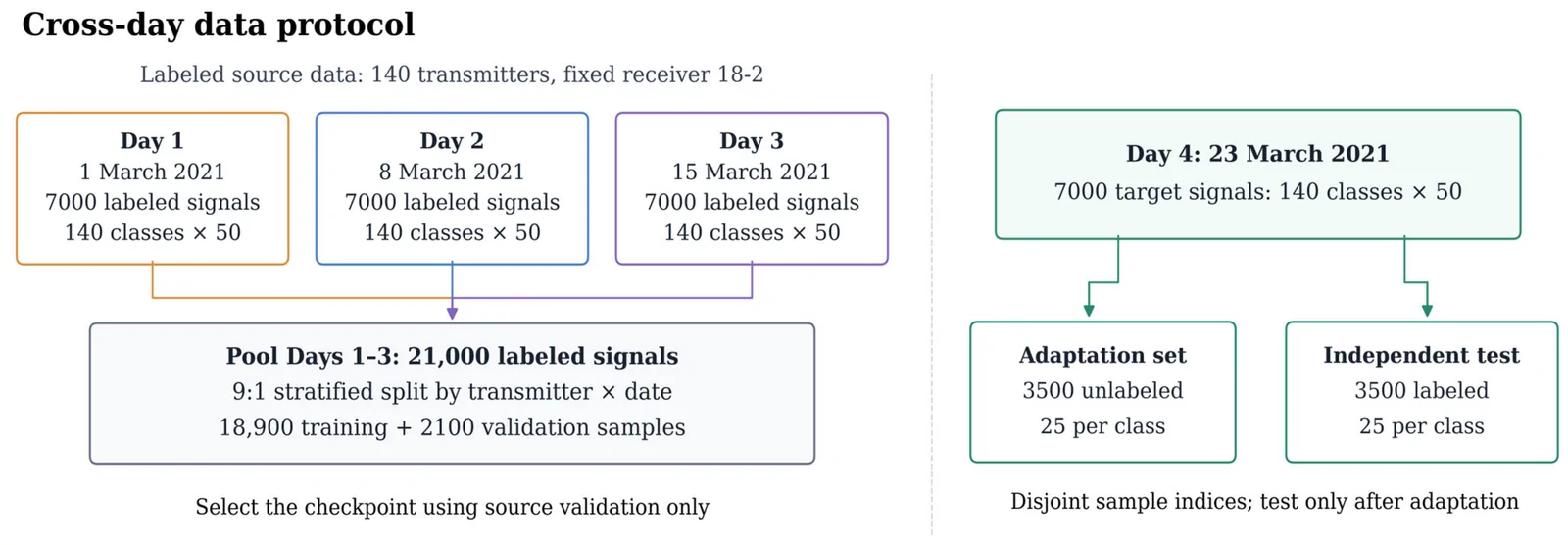
Cross-day data protocol. The three source days are pooled before a 9:1 stratified training/validation split; the Day 4 adaptation and test sets are disjoint.

Because ManyTx is not perfectly balanced, we first performed a model-independent data-availability analysis. Based only on four-day completeness, receiver 18-2 contains 140 transmitters with all 50 samples on every day in both unequalized and equalized versions, the largest number among the available receivers. No classification accuracy or Day 4 label was used during selection. To fix the receive chain and focus on date shift, the formal experiments used only the equalized signals from receiver 18-2. Each of the 140 classes included 50 samples for each date, giving 7000 samples per day and 28,000 in total. Table 3 summarizes the data and protocol, and Figure 1 illustrates the cross-day split.

Each I/Q sequence was independently normalized according to its mean power. Let the lth sample be $x_{l}=[I_{l},Q_{l}]$, and let the sequence length be *L* = 256. The normalized sequence is:

(1) $${\hat{x}}_{l}=\frac{x_{l}}{\max\left(\sqrt{\frac{1}{L}\sum_{t=1}^{L}\left(I_{t}^{2}+Q_{t}^{2}\right)},10^{-8}\right)}$$

This operation reduces received-power scale differences while preserving the normalized waveform and phase structure. All arrays were converted to float32 and transposed from an array of shape (256, 2) to one of shape (2, 256) before being passed to the one-dimensional convolutional network. After the three source days were merged, a 9:1 stratified split was performed according to the “device label × date” combination, ensuring that each device contributed the same proportion of samples from every source date to the training and validation sets. The source checkpoint was selected solely by source-validation accuracy.

Each device had exactly 50 signals on Day 4. According to the original sample indices, the first 25 samples of every class were written to the unlabeled adaptation file and the remaining 25 to the independent test file; their sample_indices did not overlap. The adaptation file stores only signals, sample_indices, tx_names, capture_date, receiver, and version, and contains no labels. Test labels were read only after all adaptation epochs had finished, for a single final evaluation. This implementation-level isolation prevented the adaptation script from directly using target labels and provides a reproducible basis for final evaluation, while external blind testing remains a stronger validation option.

The source model is a one-dimensional residual network with approximately 1.86 million parameters. Its input is a two-channel I/Q sequence. A stem convolution with kernel size 11 and stride 2 maps the input to 64 channels, followed by six residual blocks that extract multiscale temporal features over three stages with 64, 128, and 256 channels. Each residual block contains two convolutional layers with kernel sizes 7 and 5, batch normalization, ReLU, and a shortcut connection; stride-2 downsampling is used between stages. Global adaptive average pooling produces a 256-dimensional feature vector, and a linear layer outputs 140-class logits after 0.3 dropout. The architecture is summarized in Table 4. Residual connections follow the basic ResNet concept [37], while the kernels and channel configuration are designed for I/Q sequences of length 256.

The source model was trained with cross-entropy loss and 0.1 label smoothing using AdamW [38], an initial learning rate of 1 × 10^−3^, weight decay of 1 × 10^−4^, and a batch size of 256. The learning rate followed a 30-epoch cosine-annealing schedule. Training was capped at 30 epochs and stopped early if source-validation accuracy did not improve for eight consecutive epochs; the best source-validation checkpoint was retained. The three random seeds independently controlled the source training/validation split, parameter initialization, mini-batch order, and adaptation-stage randomness.

## 3. SF-CBPA Method and Experimental Design

### 3.1. Overall SF-CBPA Method

SF-CBPA adaptation began from the same source-model checkpoint and did not reload source-domain I/Q data. The unlabeled target-day adaptation set was used to calibrate BN statistics, generate balanced pseudo-labels, and update the feature extractor. The classifier remained frozen throughout to reduce unconstrained drift of the source-class decision boundaries during target self-training. The independent test set was completely isolated from the adaptation set.

We first loaded the source model and cleared the source-domain running mean and variance of every BatchNorm1D layer. The network was switched to training mode, and the unlabeled target adaptation set was traversed without gradient computation to re-estimate target-domain statistics using cumulative averaging. This step is equivalent to AdaBN calibration over the entire target adaptation set [32] and did not update convolutional weights or classifier parameters. Standalone AdaBN was also included as a baseline.

The calibrated network initialized the student parameters θ and the teacher parameters $\bar{\theta }$. For each target adaptation sample, the teacher produced logits $z_{i}$ and probabilities $p_{i}=\mathrm{softmax}(z_{i})$. Directly using argmax as the pseudo-label would cause initial cross-day errors to concentrate samples in a few classes. We therefore applied Sinkhorn normalization to the teacher’s class scores and solved for an entropy-regularized balanced assignment matrix $Q\in R^{N\times C}$:

(2) $$Q^{*}={arg\;min}_{Q}\left[-\sum_{i=1}^{N}\sum_{c=1}^{C}Q_{ic}\log p_{ic}-\varepsilon H(Q)\right]$$

where each sample has assignment mass 1 and each class has total assignment mass approximately *N*/*C*, i.e.,:

(3) $$Q1_{C}=1_{N},\;\;Q^{⊤}1_{N}=\frac{N}{C}1_{C}$$

The uniform class marginal in Equation (3) is consistent with the closed-set protocol because every class contributes the same number of unlabeled samples. Sinkhorn numerically solves this constrained assignment [33], and the balanced labels are derived directly from teacher classification scores without introducing dynamic target prototypes. The pseudo-label is ${\hat{y}}_{i}={arg\;max}_{c}Q_{ic}$. After each student-model update, the teacher parameters are updated by exponential moving average.

For reproducibility, the entropy parameter *ε* was fixed at 0.50. Sinkhorn normalization used exactly 50 alternating row and column normalization iterations over the complete adaptation set; no convergence tolerance or early-stopping criterion was used.

(4) $$\theta_{\mathrm{teacher}}\leftarrow m\theta_{\mathrm{teacher}}+\left(1-m\right)\theta_{\mathrm{student}},\;\;m=0.99$$

The EMA teacher smooths short-term gradient noise and supplies more stable pseudo-labels to subsequent epochs [36].

Balanced soft assignment guarantees the overall class marginal but does not ensure that every pseudo-label is reliable. We define the teacher’s maximum class probability as the confidence score $s_{i}=\underset{c}{\max}p_{ic}$, rank samples within each predicted class, and retain only the top proportion ρ=0.8. Let *S* denote the selected set. Unlike a global threshold, this strategy prevents easily recognized classes from occupying most of the training samples while preserving relatively reliable samples from difficult classes. Approximately 2800 of the 3500 target signals were selected in each epoch.

During adaptation, the classifier $g_{s}$ was frozen, and only the student feature extractor $h_{\theta }$ was updated. Pseudo-label cross-entropy was applied to the selected set *S*:

(5) $$L_{\mathrm{PL}}=-\frac{1}{\left|S\right|}\sum_{i\in S}\log p_{\theta }\left({\hat{y}}_{i}|x_{i}\right)$$

Meanwhile, an information-maximization term was added to the target predictions. Let $\bar{p}=\frac{1}{N}\sum_{i=1}^{N}p_{\theta }(x_{i})$ and $H(p)=-\sum_{c=1}^{C}p_{c}\log p_{c}$. Then,

(6) $$L_{\mathrm{IM}}=\frac{1}{N}\sum_{i=1}^{N}H\left[p_{\theta }(x_{i})\right]-H(\bar{p})$$

Minimizing the first term encourages confident per-sample predictions, whereas minimizing the negative marginal entropy encourages diversity in the overall predictions. The total loss is:

(7) $$\;\;\;\;\;\;\;\;\;\;\;\;\;L=L_{\mathrm{PL}}+\lambda_{\mathrm{IM}}L_{\mathrm{IM}},\;\;\lambda_{\mathrm{IM}}=0.1$$

Adaptation used the AdamW optimizer with a learning rate of $5\times 10^{-5}$, a weight decay of $1\times 10^{-4}$, gradient-norm clipping at 5, and ten training epochs. The final EMA teacher was used for independent testing.

From an optimization perspective, each epoch first used the EMA teacher to regenerate soft assignments over the complete adaptation set and then trained the student network. Pseudo-labels were therefore not balanced independently within each mini-batch; instead, they used class statistics from the full set of 3500 target samples. This batch design is appropriate for periodic edge updates and has manageable computational and memory costs. Streaming operation would require a queue or an online marginal estimator to maintain a similar constraint.

### 3.2. Design Rationale and Leakage Prevention

The components of SF-CBPA form a dependency chain that first restores statistics, then stabilizes the teacher, constrains class marginals, and finally optimizes target features. AdaBN first reduces the overall shift in shallow activation statistics so that the teacher’s initial predictions are not completely distorted. Freezing the classifier preserves source-class semantics and prevents unlabeled target optimization from moving both features and decision boundaries simultaneously. The EMA teacher reduces pseudo-label variation across individual mini-batches; Sinkhorn-balanced assignment controls global class coverage; and within-class top-80% selection performs reliability filtering after balancing. If a global threshold were applied before balancing, infrequently predicted classes could disappear during filtering and could not be recovered by subsequent balancing.

The benefit of the class-balancing constraint depends on the target class prior. In the present protocol, every class contributes exactly 25 unlabeled samples, which provides an explicit empirical basis for the uniform marginal used in Equation (3). For a known nonuniform class prior, that equation can instead prescribe class-specific assignment masses proportional to the prior. If the prior is unknown, strongly imbalanced, or contains absent classes, a fixed uniform constraint can force incorrect assignments. Practical extensions would therefore require a prior estimated from historical activity, smoothed target predictions, or a partial/open-set mechanism. These conditions are outside the controlled balanced protocol evaluated in this study and are treated as limitations rather than as empirically validated capabilities.

To prevent information leakage from the target test set, the code separates $D^{\mathrm{ta}}$ and $D^{te}$ at the file level. The adaptation file contains no labels key, and the adaptation script opens the test file only after all ten training epochs have finished and the final model has been saved. Hyperparameters were not selected using independent-test accuracy; all ablation experiments used fixed epochs, learning rate, selection ratio, and random seeds. The source-model checkpoint was selected solely according to source-validation accuracy. This design strengthens protection against inadvertent leakage by keeping target labels outside the adaptation process; external blind testing would provide an additional level of validation.

### 3.3. Comparison Methods, Evaluation Metrics, and Implementation Settings

All comparison methods used the same source-model architecture and Day 4 split. Source-only, AdaBN, TENT-style, SHOT, and SF-CBPA were evaluated with random seeds 42, 123, and 2026.

Source-only: no target adaptation was performed; the source model trained on the first three days was directly evaluated on the independent Day 4 test set.

AdaBN: the unlabeled target adaptation set was used only to re-estimate the running statistics of all BatchNorm layers; no network weights were updated [32].

TENT-style: prediction entropy was minimized on the target adaptation set, and only BatchNorm affine parameters were updated; 4480 parameters were adaptable [12].

SHOT: the source classifier was frozen and the feature extractor was adapted using information maximization and pseudo-label self-training [11]. Pseudo-labels were refreshed once per epoch through two cosine-centroid refinement steps. The official objective weights (0.3 for pseudo-label cross-entropy and 1.0 for information maximization) were retained; the effective feature learning rate was 1 × 10^−3^ with SGD, momentum 0.9, Nesterov acceleration, and the official polynomial decay schedule. The common batch size of 256 and ten-epoch adaptation budget were used for protocol matching.

SF-CBPA: the proposed method, comprising AdaBN initialization, a frozen classifier, an EMA teacher, Sinkhorn class-balanced pseudo-labeling, within-class top-80% selection, and an auxiliary information-maximization term.

The nested ablation evaluated AdaBN + PL, AdaBN + PL + IM, AdaBN + CBPL, and the complete SF-CBPA. All pseudo-label variants in that table retained the EMA teacher. PL used ordinary softmax pseudo-labels with global top-80% confidence selection, whereas CBPL denotes Sinkhorn-balanced assignment followed by within-class top-80% selection. IM denotes information maximization. Because the nested comparison changes both balancing and selection, its CBPL gain is interpreted at the block level. A separate one-factor-at-a-time table isolates EMA, Sinkhorn, within-class selection, and IM while keeping the other settings fixed.

We report accuracy, macro-averaged precision, macro-averaged recall, and macro-F1. Because the independent test set contained 25 samples from every class, accuracy and macro-averaged recall are numerically identical; both are retained for comparison with general classification literature. The main results are reported as the mean ± sample standard deviation over random seeds 42, 123, and 2026.

For each random seed, an exact McNemar test [39] was applied to paired predictions on the same independent test samples. Let *b* be the number of samples classified correctly by the baseline but incorrectly by SF-CBPA, and *c* the number classified incorrectly by the baseline but correctly by SF-CBPA. The null hypothesis is that the two methods have equal marginal error rates. In addition, a class-stratified paired bootstrap with 2000 resamples was conducted for seed 123 to estimate 95% confidence intervals for the gains in accuracy and macro-F1 [40]. The paired design preserved the dependence between model predictions on the same test samples.

Experiments were conducted using Python 3.12, PyTorch 2.13.0+cu126, and an NVIDIA GeForce RTX 2060 GPU with 6 GB of memory. On a Windows platform, DataLoader used num_workers = 0 and a batch size of 256. For reproducibility, the Python, NumPy, and PyTorch random seeds were fixed, deterministic cuDNN was enabled, and benchmarking was disabled. Every adaptation variant restarted from the original source model for its corresponding random seed and was never trained from a previous adaptation result. Table 5 summarizes the principal hyperparameters.

The adaptation parameters were fixed before independent-test evaluation and were not selected using Day 4 test labels. The EMA momentum *m* = 0.99 provides conservative temporal smoothing; ρ=0.8 discards the lowest-confidence 20% within each predicted class while retaining most samples; $\lambda_{\mathrm{IM}}=0.1$ keeps information maximization auxiliary to pseudo-label cross-entropy; and ten epochs impose a bounded common adaptation budget. Reviewer-motivated post hoc sensitivity at seed 123 evaluated ρ={0.6,0.8,1.0} and $\lambda_{\mathrm{IM}}=\{0,0.1,0.2\}$. These settings are reported as design choices, not claimed optima.

To isolate the effect of the adaptation strategy, all five comparison methods used the same ResNet1D source model and the same Day 4 split. Source-only updated no parameters; AdaBN updated only running statistics; TENT-style updated 4480 BN affine parameters; and SHOT and SF-CBPA froze the classifier while updating the feature extractor. Although the numbers of adaptable parameters differ, the final inference architecture is identical. The comparison therefore reflects the combined effect of the adaptation strategy and its update freedom. The complexity analysis reports the corresponding differences in adaptation cost.

Results for all three random seeds are reported in full. Seed 123, the predesignated middle-numbered seed, was used for ablation, bootstrap, and sample-size sensitivity; its SF-CBPA accuracy was 62.94%, compared with 64.74% for seed 42. The main conclusions rely on the three-seed mean ± sample standard deviation, while single-seed experiments explain component behavior.

The statistical analysis separates two questions: whether predictions change and how large the metric change is. The exact McNemar test uses paired binary correct/incorrect outcomes to determine whether marginal error rates differ; the paired bootstrap provides uncertainty intervals for the gains in accuracy and macro-F1. Given the three training seeds, sample-level paired statistics complement the mean and standard deviation, while a low-power parametric test over seed means was not used.

These settings ensured that differences among methods arose primarily from the adaptation mechanisms rather than from the source model, test split, or selective result reporting. In particular, all adaptation variants restarted from the same source checkpoint for the corresponding random seed, and Day 4 test labels were not involved in training, early stopping, or hyperparameter selection. By fully reporting all three random seeds, fixing the ablation path, and using paired sample-level statistics, the experimental design controls source-model initialization variability, target-split differences, and selective-reporting risk.

## 4. Results

### 4.1. Overall Performance and Statistical Significance

**Figure 2:**
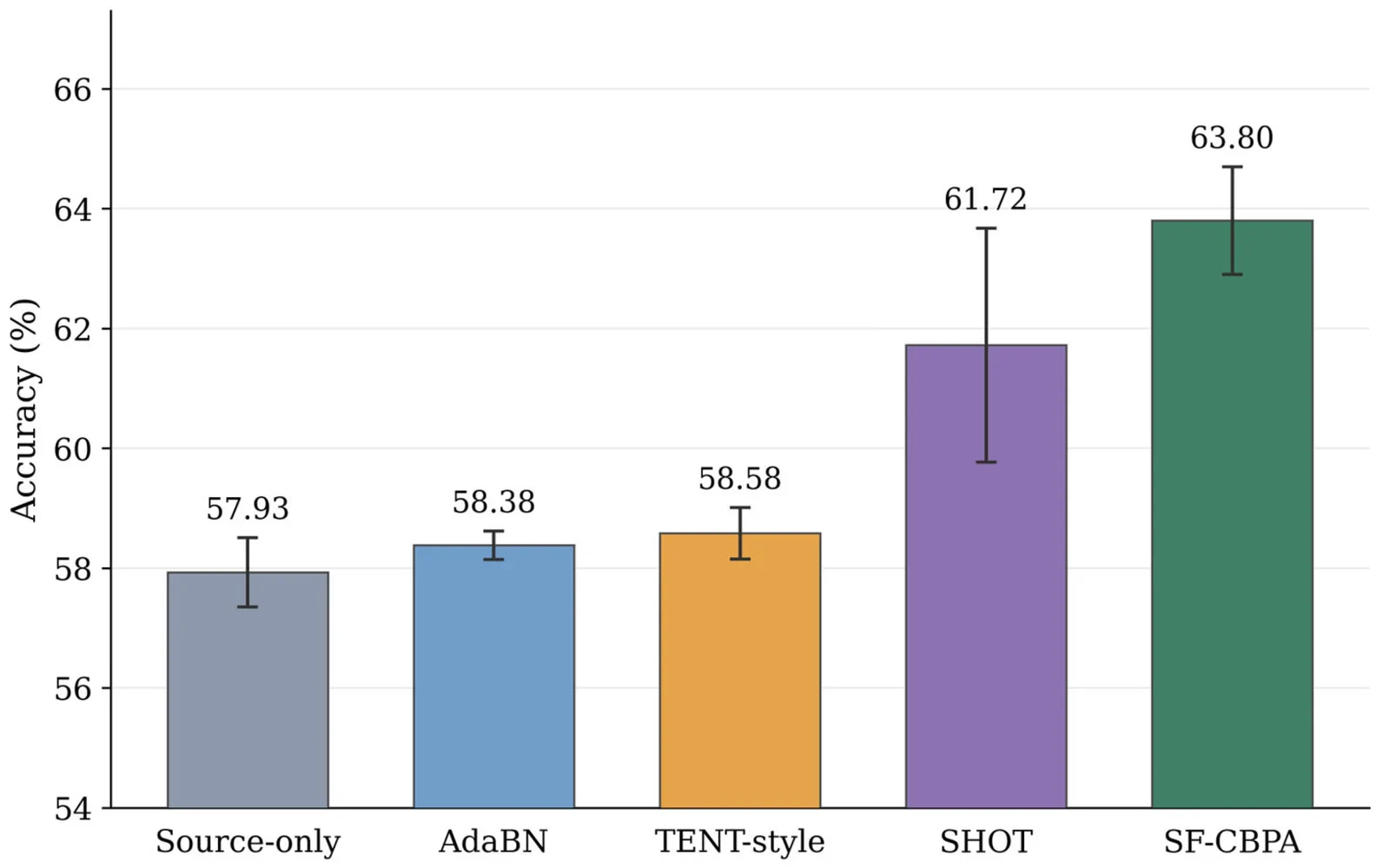
Three-seed mean accuracy of five methods on the independent Day 4 test set. Error bars indicate the sample standard deviation across the three seeds.

**Figure 3:**
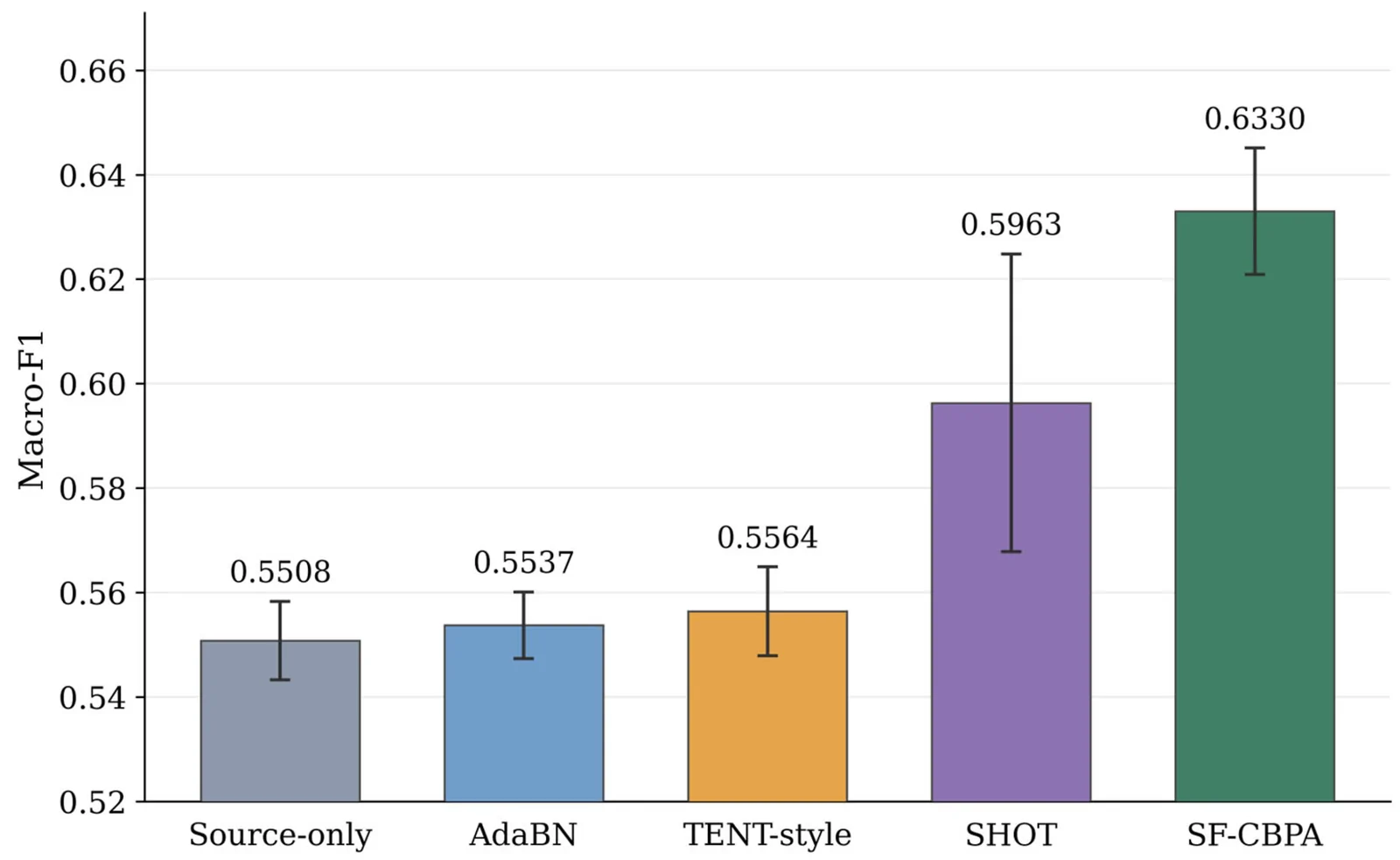
Three-seed mean macro-F1 of five methods on the independent Day 4 test set. Error bars indicate the sample standard deviation across the three seeds.

Table 6 presents the mean and sample standard deviation of five methods over three random seeds; Figure 2 and Figure 3 show accuracy and macro-F1. Source-only reached 57.93% mean accuracy, AdaBN 58.38%, and TENT-style 58.58%. SHOT improved the result to 61.72 ± 1.95% accuracy and 0.5963 ± 0.0285 macro-F1, demonstrating the value of adapting the feature extractor while preserving the source classifier. SF-CBPA reached 63.80 ± 0.90% accuracy and 0.6330 ± 0.0121 macro-F1, exceeding SHOT by 2.08 percentage points in mean accuracy under the matched protocol.

SF-CBPA outperformed Source-only, AdaBN, TENT-style, and SHOT in mean accuracy by 5.87, 5.42, 5.22, and 2.08 percentage points, respectively. Its macro-F1 exceeded SHOT by 0.0367. Because the SHOT sample’s standard deviation was 1.95 percentage points, compared with 0.90 for SF-CBPA, the three-seed result also indicates lower initialization sensitivity for the proposed method in this experiment. No significance claim is made for the descriptive SHOT difference, because the paired statistical tests in Table 7 were prespecified for the original baselines.

Viewed in terms of error rate, Source-only had a mean error of 42.07%, whereas SF-CBPA reduced this to 36.20%, corresponding to a relative error reduction of 13.95%. Relative to the 41.42% error of TENT-style, the reduction is 12.60%. On this difficult cross-day task, where performance remained below 70%, the error-rate perspective shows that a 5–6 percentage-point gain is practically meaningful. The remaining 36.20% error positions SF-CBPA as a component of multifactor authentication or risk screening rather than a standalone high-security authenticator.

**Figure 4:**
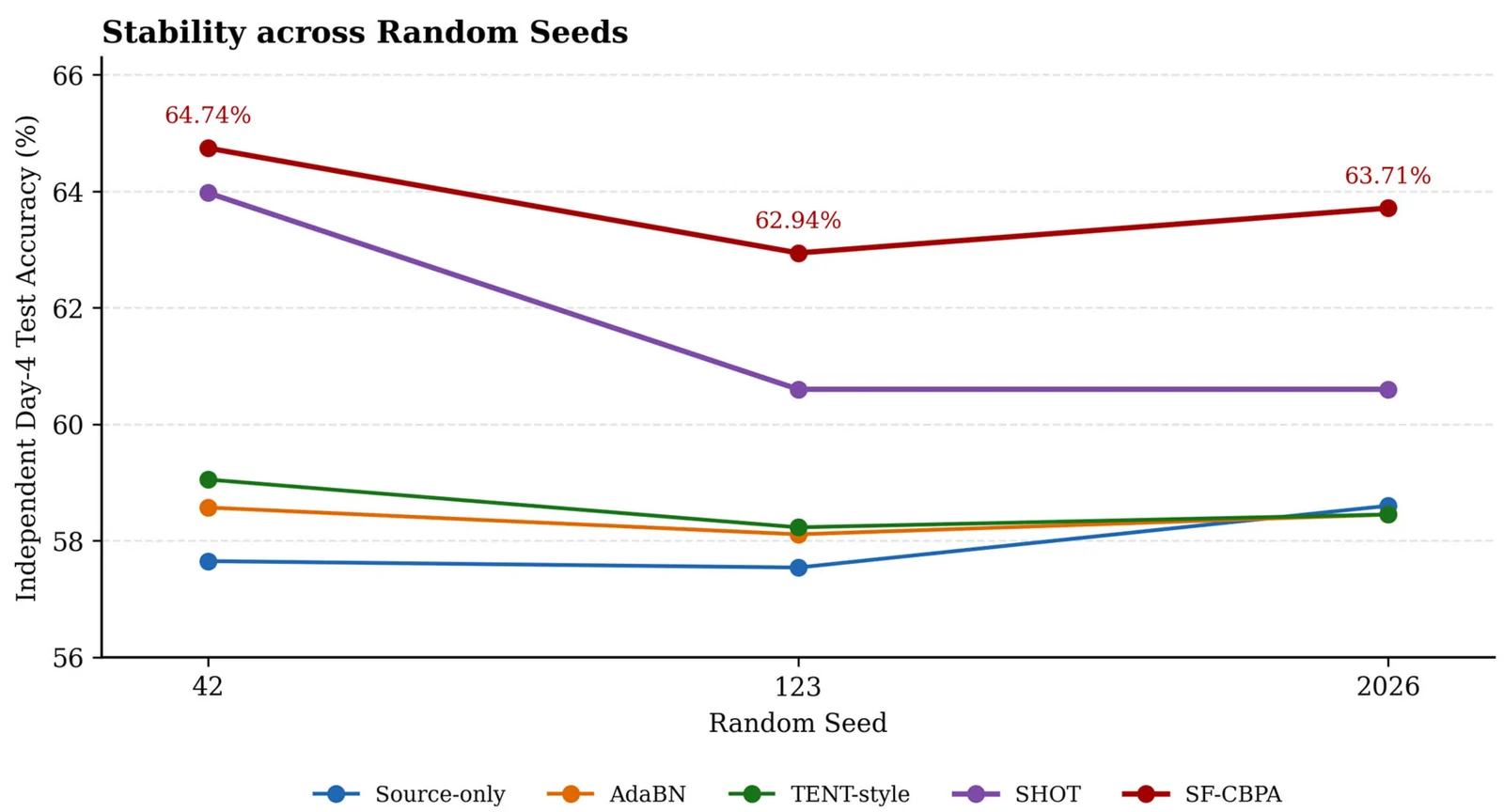
Matched accuracy of five methods over three random seeds.

Figure 4 places all five methods for each random seed in the same coordinate system. SHOT reached 63.97% for seed 42 but 60.60% for seeds 123 and 2026; SF-CBPA reached 64.74%, 62.94%, and 63.71%, respectively. Thus, SF-CBPA was higher for every matched source checkpoint, while SHOT remained substantially stronger than the lighter baselines. The variation across seeds reinforces the need to report means and sample standard deviations rather than a single favorable run.

Table 7 reports exact McNemar test results comparing SF-CBPA with the three original baselines for every random seed. In all comparisons, *c* is substantially larger than *b*, indicating that SF-CBPA corrected far more erroneous predictions than it introduced. The largest *p*-value relative to Source-only is 3.05 × 10^−27^, and the largest *p*-value relative to TENT-style is 4.09 × 10^−31^, both far below 0.05. The paired evaluation on the same 3500 test samples controls test-sample resampling variability.

**Figure 5:**
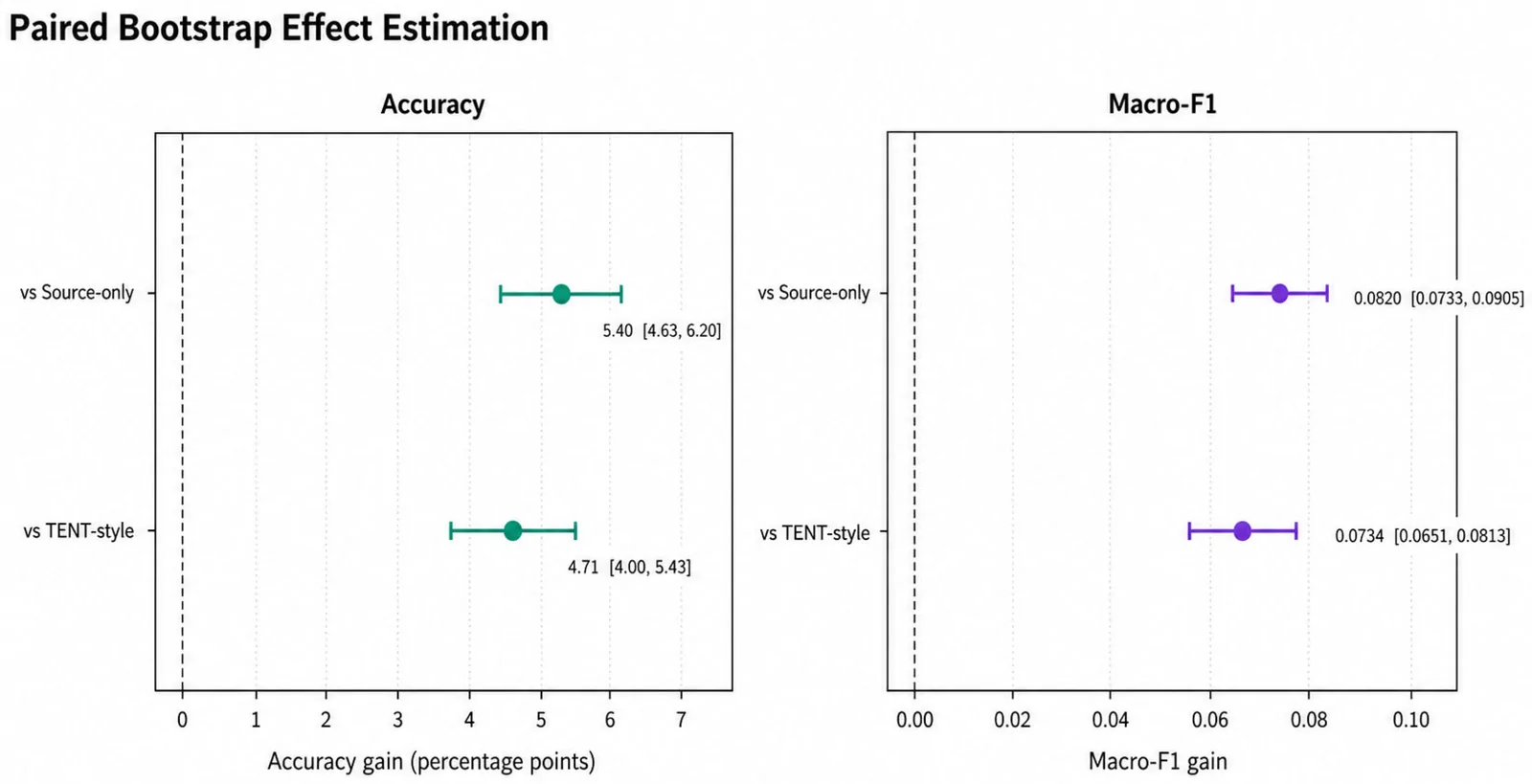
Paired-bootstrap effect estimates for seed 123.

Figure 5 presents the paired-bootstrap effect estimates for seed 123. For this seed, the 95% paired-bootstrap interval for the accuracy gain of SF-CBPA over Source-only was [4.63, 6.20] percentage points, and the macro-F1 gain interval was [0.0733, 0.0905]. The corresponding intervals relative to TENT-style were [4.00, 5.43] percentage points and [0.0651, 0.0813]. None of the four intervals crosses zero, consistent with the McNemar results.

McNemar and bootstrap analyses answer complementary questions. The former uses the asymmetry between *b* and *c* to test whether two classifiers have different error rates on the same samples; the latter directly quantifies the plausible range of metric gains. The confidence-interval lower bounds of 4.63 and 4.00 percentage points complement the small McNemar *p*-values by demonstrating an observable effect size.

In engineering terms, SF-CBPA reduced the mean error rate from 42.07% for Source-only to 36.20%, which corresponds to a relative error reduction of 13.95%; the relative reduction compared with TENT-style was 12.60%. The absolute accuracy gains, macro-F1 gains, paired confidence intervals, and relative error reductions therefore provide consistent evidence of effectiveness. Nevertheless, the remaining 36.20% error rate indicates that the method is better suited to auxiliary authentication or risk screening than to standalone high-security identity decisions.

### 4.2. Ablation, Sample-Size Sensitivity, and Class-Level Performance

**Figure 6:**
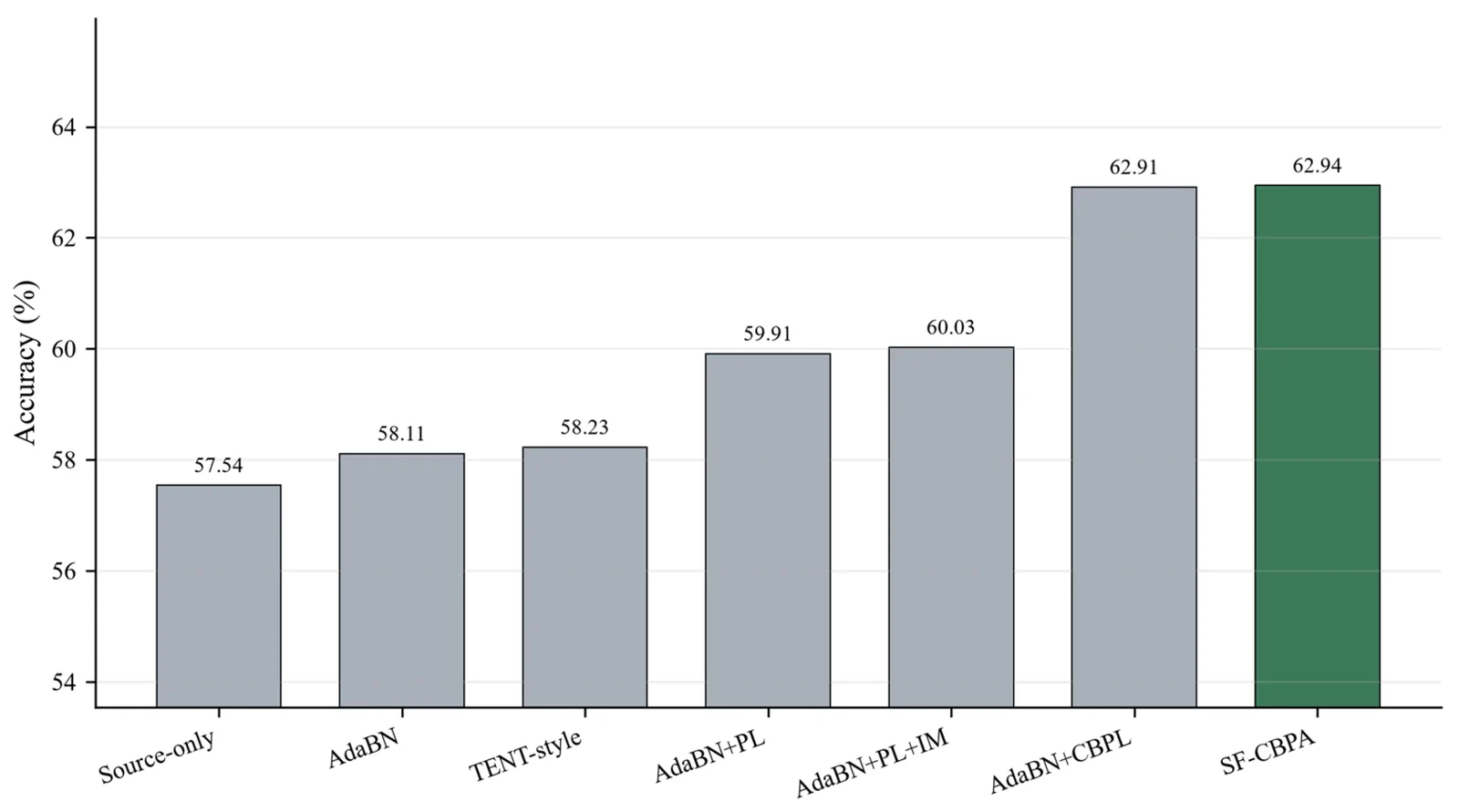
Accuracy of the nested SF-CBPA ablation on the independent Day 4 test set (seed 123). Gray bars denote the comparison methods and intermediate variants; the green bar denotes the complete SF-CBPA method.

The ablation study used seed 123 and fixed the source model, data split, number of adaptation epochs, and optimizer. Results are shown in Table 8 and Figure 6. AdaBN improved on Source-only by 0.57 percentage points. Adding ordinary pseudo-labeling to AdaBN (AdaBN + PL) raised the accuracy to 59.91%, indicating that target self-training can exploit part of the high-confidence structure. Adding IM produced only a further 0.12-point gain. Replacing ordinary pseudo-labels with class-balanced pseudo-labels (AdaBN + CBPL) increased accuracy substantially to 62.91%, 3.00 percentage points above AdaBN + PL, while improving macro-F1 by 0.0550. The complete SF-CBPA reached 62.94%, only 0.03 points above CBPL.

A supplementary three-seed comparison yielded 63.70 ± 0.83% for SF-CBPL and 63.80 ± 0.90% for the complete SF-CBPA. Information maximization therefore contributed only +0.10 percentage points in mean accuracy and +0.0009 in macro-F1. We consequently identify class-balanced pseudo-labeling as the core effective component and treat IM as a lightweight auxiliary regularizer rather than presenting it as a separate primary contribution.

Table 8 follows a nested block structure. Its +3.00-point CBPL difference measures replacement of ordinary pseudo-labeling by the combined balanced-assignment and within-class-selection block. To remove this confounding, Table 9 holds the remaining settings fixed and disables EMA, Sinkhorn, within-class selection, or information maximization one at a time.

The isolated results in Table 9 identify Sinkhorn as the dominant tested component; removing it lowered accuracy by 2.45 percentage points and macro-F1 by 0.0404. Removing within-class selection lowered accuracy by 0.23 points, and removing IM by 0.03 points. The no-EMA variant was 0.49 points higher than the full seed-123 result, so EMA is not claimed to yield an independent accuracy gain; its role is limited to temporal smoothing.

**Figure 7:**
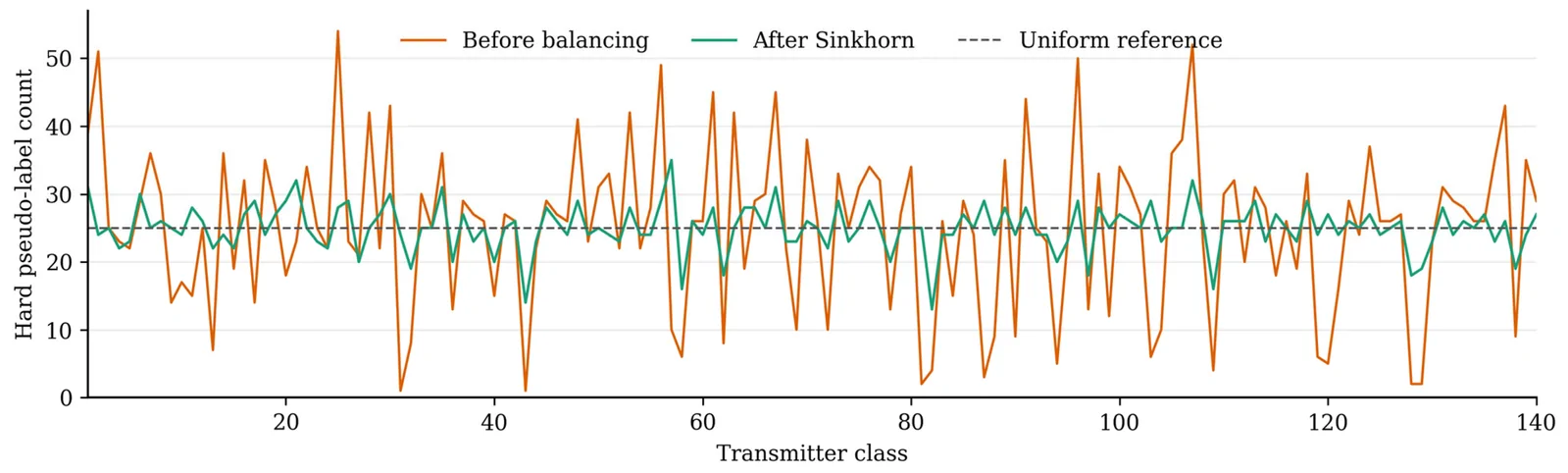
Hard pseudo-label distribution before and after Sinkhorn balancing for seed 123; the dashed line denotes the uniform count of 25.

Figure 7 directly compares hard pseudo-label counts produced from the same AdaBN-calibrated seed-123 logits. Before balancing, all 140 classes were active, but counts ranged from 1 to 54 (standard deviation 11.75; normalized entropy 0.9739). After Sinkhorn, the range narrowed to 13–35 (standard deviation 3.42; normalized entropy 0.9980). Subsequent class-wise top-80% selection retained all 140 classes with counts of 10–28. Thus, balancing markedly reduced concentration while not forcing exactly uniform hard argmax counts.

Table 10 reports the requested seed-123 sensitivity. Accuracy varies by 0.74 percentage points across ρ=0.6−1.0 and by 0.23 points across $\lambda_{\mathrm{IM}}=0-0.2$. The best tested values are ρ=0.6 and $\lambda_{\mathrm{IM}}=0.2$, but these reviewer-motivated post hoc runs were not used to replace the prespecified headline configuration. The narrow ranges indicate limited local sensitivity, not global optimality.

**Figure 8:**
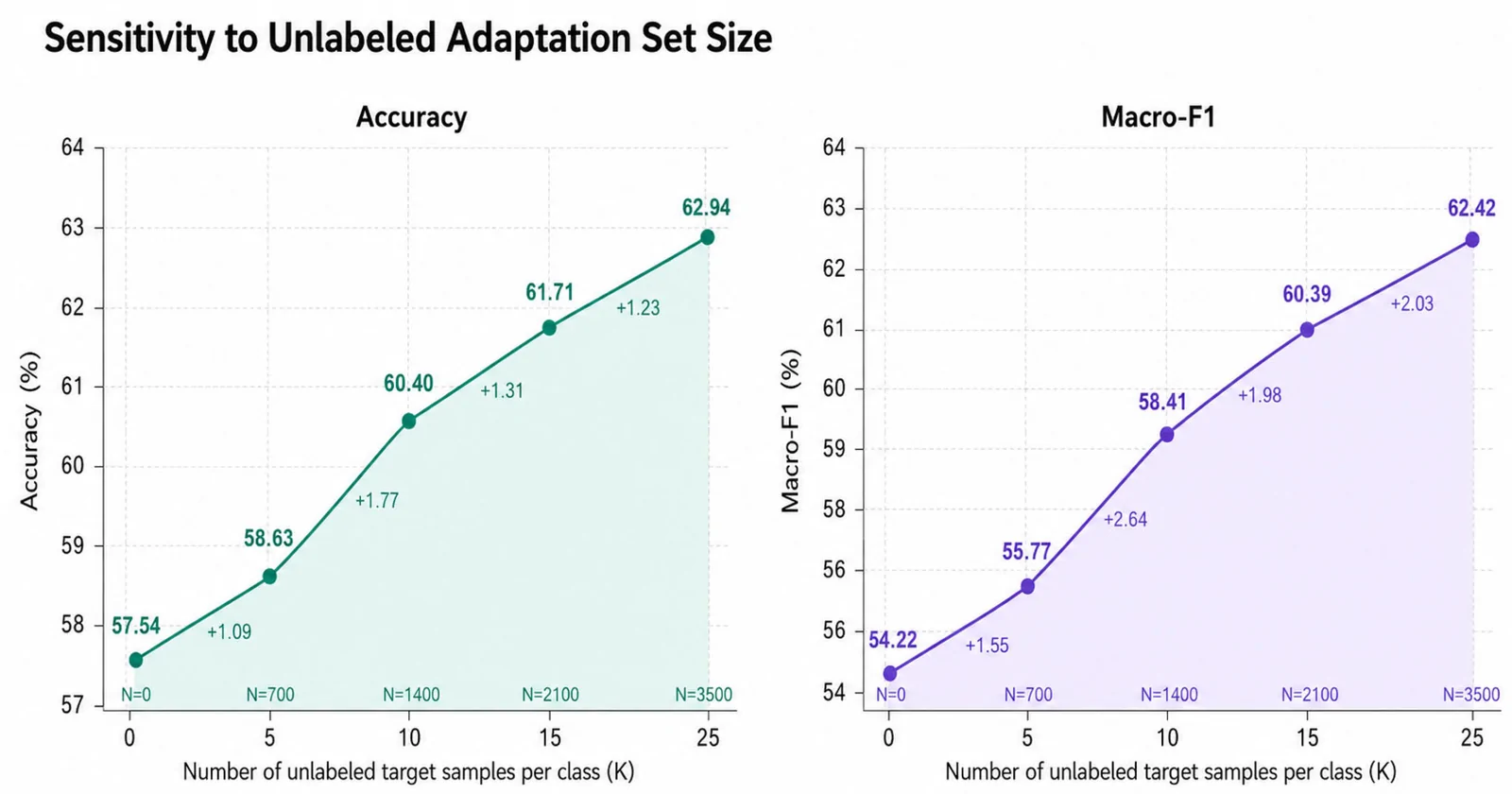
Effect of the number of unlabeled adaptation samples per class, *K*, on SF-CBPA performance (seed 123).

To evaluate the amount of unlabeled target-day data required, seed 123 was fixed and the number of adaptation samples per class, *K*, was set to 0, 5, 10, 15, and 25. *K* = 0 corresponds to Source-only; every other setting restarted from the same source model. Table 11 and Figure 8 show that accuracy increased monotonically from 57.54% to 62.94% as *K* increased, while macro-F1 increased from 0.5422 to 0.6242.

The marginal efficiency was nonlinear. Increasing *K* from 5 to 10 added 700 samples and improved accuracy by 1.77 percentage points, whereas increasing *K* from 15 to 25 added 1400 samples but improved accuracy by 1.23 points, indicating a clear decline in benefit per sample. In practice, deployment can select an operating point according to collection cost; 10–15 samples per class already recovered approximately 53–77% of the total accuracy gain achieved with 25 samples, while collecting 25 samples approached the highest performance. This operating-point analysis is specific to the present balanced closed-set protocol; imbalanced class priors require separate validation.

**Figure 9:**
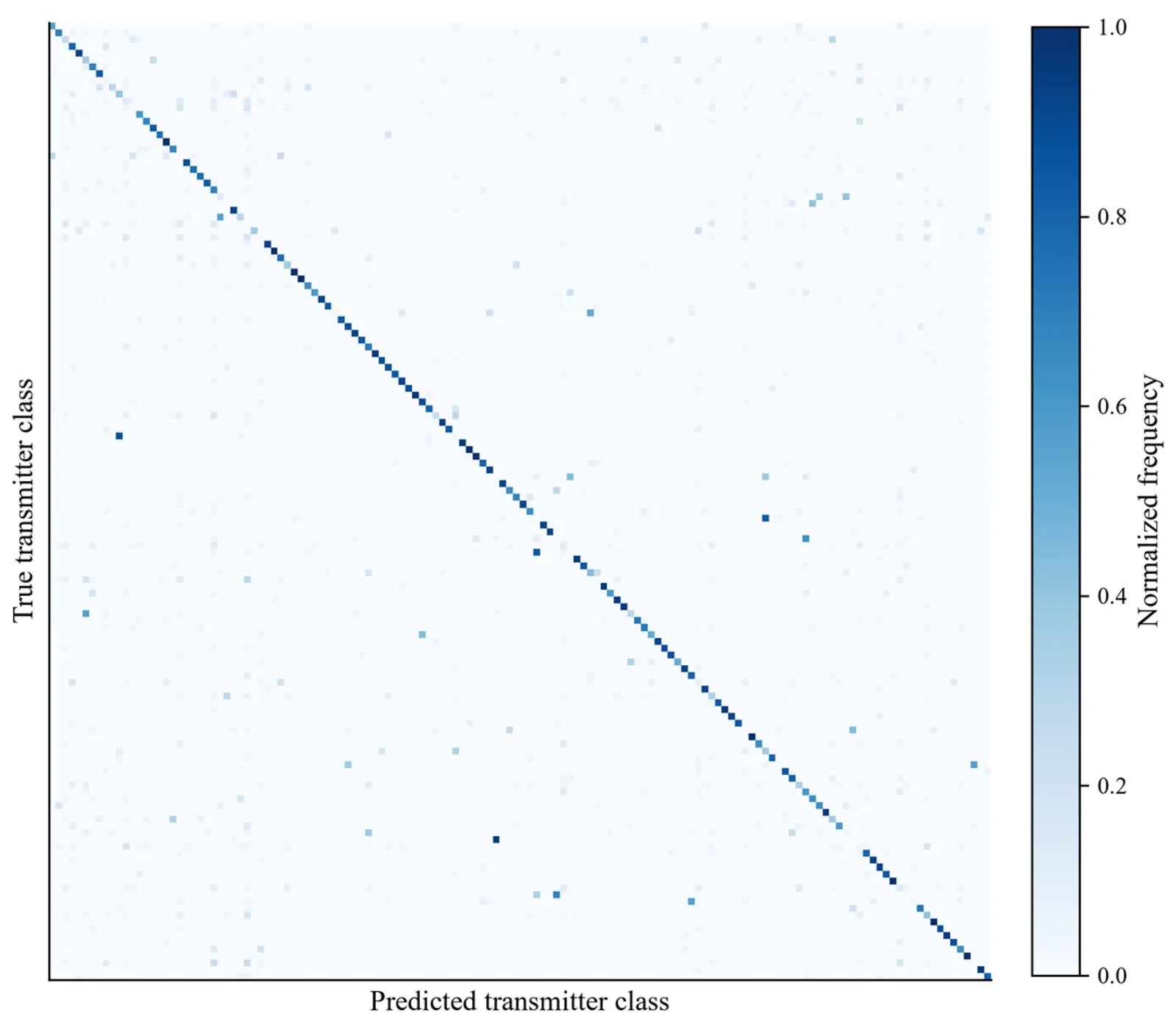
Row-normalized 140-class confusion matrix of SF-CBPA on the independent Day 4 test set (seed 123).

Figure 9 shows the row-normalized confusion matrix of the complete SF-CBPA on the independent test set for seed 123. The prominent diagonal indicates that many devices retain discriminative structure, whereas groups of off-diagonal confusion show that difficult devices remain. Macro-F1 is close to but below accuracy, motivating future device-level uncertainty, calibration, and rejection analysis.

Taken together, the block-level ablation, sample-size analysis, and confusion matrix are consistent with improved supervision of difficult classes and with gains that are not confined to a small subset of devices. As the number of target samples increases, balanced assignment and within-class ranking are supported by more stable statistics, while the marginal benefit per additional sample decreases. These observations support a bounded batch-calibration strategy under the balanced closed-set protocol; they do not by themselves establish robustness to unknown priors or missing classes.

### 4.3. Computational Complexity

Table 12 summarizes the model and adaptation complexity. The source ResNet1D contained 1,860,492 parameters and its checkpoint was approximately 7.14 MB. TENT-style updated 4480 BN affine parameters, whereas SHOT and SF-CBPA froze the classifier and updated the feature extractor. SF-CBPA additionally maintained an EMA teacher and performed full-set balanced assignment during its ten offline epochs; the final inference architecture remained unchanged.

In batched forward evaluation on an RTX 2060 with batch size 256, throughput was approximately 20,223 samples/s, corresponding to an amortized time of 0.049 ms/sample. This number reflects batched GPU throughput; end-to-end single-packet latency also includes data transfer, preprocessing, single-sample scheduling, and adaptation. Because the adapted model and source model shared the same architecture, the number of parameters used for a single inference did not increase after adaptation.

## 5. Discussion

### 5.1. Operating Mechanism and Evidence Boundaries

Table 13 maps the four research questions considered in this study to the experimental evidence and the justified scope of each conclusion. The matched SHOT comparison strengthens RQ2, while the direct distribution analysis and one-factor ablation sharpen RQ3 without implying universal optimality.

The main results and ablations jointly show that the key obstacles in large-class cross-day identification include not only shifts in overall feature means or variances, but also class-imbalanced errors produced by the source model on the target day. Ordinary pseudo-labeling selects samples by model confidence. If several classes receive higher initial target logits, they acquire more pseudo-labels and more gradient updates, producing positive feedback in which easy classes become easier while difficult classes disappear. AdaBN and TENT-style can adjust the distribution to some extent but do not explicitly control class marginals, and their mean gains are therefore below one percentage point.

SF-CBPA interrupts concentrated self-training through two constraints. For seed 123, ordinary softmax argmax labels covered all 140 classes but are strongly skewed; hard counts ranged from 1 to 54, their standard deviation was 11.75, and normalized class entropy was 0.9739. Applying Sinkhorn to the same AdaBN-calibrated logits narrowed the hard-count range to 13–35, lowered the standard deviation to 3.42, and raised normalized entropy to 0.9980. Class-wise top-80% selection retained all 140 classes with counts of 10–28. The evidence therefore supports a reduction in pseudo-label concentration, not a claim that classes were already absent before balancing.

Equation (3) provides an analytical guarantee for the soft assignment marginal; with *N* = 3500 and *C* = 140, each class receives prescribed soft mass 25. Figure 7 complements this guarantee with empirical hard-label counts. Table 9 then links the mechanism to performance; removing Sinkhorn reduced seed-123 accuracy from 62.94% to 60.49% and macro-F1 from 0.6242 to 0.5838. Removing within-class selection caused a smaller decrease to 62.71% accuracy, while removing IM changed accuracy by only −0.03 points.

Information maximization contributed only 0.03 percentage points for seed 123 and 0.10 points on average over three seeds, so it remains an auxiliary term. The no-EMA variant reached 63.43%, 0.49 points above the full seed-123 result. We therefore do not claim that EMA independently improves accuracy; it is retained as a conservative temporal-smoothing mechanism whose value requires more seeds and momentum sensitivity analysis. Sinkhorn class-marginal control is the component most directly supported by both the distribution diagnostic and the isolated performance ablation.

### 5.2. Practical Deployment and Authentication Requirements

From a deployment perspective, the source-free setting reduces the need for long-term retention and cross-node transmission of source I/Q data. A device or edge node needs to retain only the approximately 7.14 MB source model and can perform offline calibration using unlabeled signals collected on the target day. Sample-size sensitivity shows that 5–15 target signals per class already yield observable gains, making periodic batch updates feasible. After adaptation, the inference architecture remains unchanged and the number of online forward-pass parameters does not increase.

The mean top-1 accuracy of 63.80% is scientifically meaningful relative to the matched baselines, but the remaining 36.20% error rate is not acceptable for standalone high-security authentication. Moreover, closed-set multiclass accuracy cannot be converted directly into an authentication operating threshold. Deployment decisions require application-specific false-acceptance and false-rejection rates, receiver-operating-characteristic or equal-error-rate analysis, confidence calibration, rejection of unknown devices, and a defined threat model. According to the evidence available here, SF-CBPA is best viewed as an auxiliary factor for multifactor authentication, risk scoring, or anomaly screening, with secondary verification for low-confidence or out-of-distribution inputs.

Under the present assumptions, a feasible engineering workflow contains five stages. First, a source ResNet1D is trained and validated on multiple historical dates in a controlled environment, and only the checkpoint and class mapping are distributed. Second, after detecting temporal, environmental, or performance drift, the deployment node accumulates a small number of unlabeled I/Q segments for each registered device. Third, during an isolated adaptation window, the node re-estimates BN statistics and runs ten epochs of SF-CBPA while the previous model continues serving online requests. Fourth, the adapted model passes unlabeled quality gates such as class coverage, mean confidence, and change in prediction entropy. A production system can define application-specific gate thresholds and retain a rollback mechanism. Fifth, after validation, the inference model is atomically replaced and the rejection rate and prediction distribution continue to be monitored.

### 5.3. Generalizability, Prior-Shift Robustness, and Limitations

Fixing receiver 18-2 removes receive-chain variation as a major confounder and strengthens internal validity for the specific question of cross-day drift. The same choice limits external validity; the experiment does not establish invariance to receiver hardware, distance, mobility, protocol, or front-end change. The consistent mechanism evidence, the matched SHOT comparison, and the use of posterior-based rather than receiver-specific handcrafted features provide scientific motivation for broader testing, but they are not empirical proof of cross-dataset generalization.

This workflow emphasizes background batch updating rather than backpropagating after every arriving packet. Sinkhorn balancing requires an estimate of the target class marginal, and a very small online batch could mistake incidental traffic differences for missing classes. If the system cannot ensure that every registered device appears within the adaptation window, the fixed uniform prior should be replaced by historical activity rates, smoothed priors, or unknown-class detection.

This study has several limitations. First, it used only WiSig-ManyTx, one fixed receiver, and equalized signals; cross-receiver, cross-distance, unequalized, mobile, and different-protocol settings remain untested. Second, the target adaptation set was a strictly balanced 140-class closed set. A uniform Sinkhorn prior may force erroneous assignments when activity is imbalanced or classes are absent; this revision does not claim robustness to prior shift or partial/open-set conditions. Third, the adaptation was a 3500-sample batch process rather than streaming test-time adaptation. Fourth, the Day 4 adaptation and test sets were disjoint by sample index but originated from the same collection date. Fifth, only general source-free/test-time baselines, including SHOT, have been evaluated under this exact ManyTx-140 protocol; more RF-specific comparisons remain necessary. Finally, the seed-123 sensitivity results cover only ρ and $\lambda_{\mathrm{IM}}$ over narrow grids; *m*, epoch count, and cross-dataset hyperparameter transfer require further study.

Future research should prioritize three directions. First, the fixed uniform class prior should be extended to a nonuniform prior that can be estimated online, while handling absent devices and newly appearing devices. Second, joint transfer across dates and receivers should be investigated to evaluate external validity under different receive chains, distances, SNRs, and protocols. Third, streaming or continual source-free adaptation should be developed, using queues, memory banks, and drift detection to control catastrophic forgetting and the accumulation of erroneous pseudo-labels. Recent work on source-free cross-receiver adaptation [26], dynamic distribution alignment [30], receiver-agnostic identification [28,29], feature disentanglement [31], and open-set recognition [21] provides useful technical routes for these extensions. Table 14 summarizes the principal threats to validity, current controls, and further validation required.

## 6. Conclusions

This study addresses cross-day distribution shift in large-scale WiFi transmitter identification through SF-CBPA. Under the balanced, closed-set, fixed-receiver WiSig-ManyTx protocol, SF-CBPA achieved 63.80 ± 0.90% mean accuracy and 0.6330 ± 0.0121 macro-F1 across three seeds. The matched SHOT implementation reached 61.72 ± 1.95% and 0.5963 ± 0.0285, respectively. Direct pseudo-label analysis and component-isolated ablation show that Sinkhorn substantially reduced class-count concentration and produced the clearest single-component gain; within-class selection was modestly beneficial, IM was auxiliary, and EMA had no independent accuracy benefit for seed 123. Sensitivity over the tested ρ and $\lambda_{\mathrm{IM}}$ ranges was limited to 0.74 and 0.23 accuracy points. The method is therefore supported as a balanced closed-set cross-day adaptation approach, not as a standalone high-security authenticator or as a validated solution to imbalanced, missing-class, cross-receiver, open-set, or streaming conditions.

## Figures and Tables

**Table 1:** Positioning of this work relative to representative studies.

Research	Key Tasks/Data	Source Data Available During Target Domain Adaptation	Core Mechanism	Key Differences from This Article
WiSig [7]	Large-scale WiFi RFFI	Not applicable	Dataset and cross-Rx/cross-day evaluation	Provides the problem and data foundation; no source-free adaptation
Alhazbi et al. [8]	Long-term RFFI stability	Not applicable	Analysis of temporal drift and power-cycle effects	Focuses on causes and preprocessing rather than ManyTx-140 target adaptation
TENT [12]	General test-time adaptation	No	Target-entropy minimization and BN affine updating	Does not explicitly address pseudo-label collapse with many classes
SHOT [11]	General source-free domain adaptation	No	Frozen classifier, IM, and pseudo-labeling	Not designed for cross-day RF fingerprints or balanced class marginals
Wu et al. [9]	Cross-day SEI; ManySig and LoRa	Yes	Adversarial alignment, pseudo-labeling, and cross-domain contrastive learning	UDA setting; different dataset and class scale
This work	ManyTx; 140-class cross-day WiFi RFFI	No	AdaBN + EMA + Sinkhorn CBPL + within-class selection	Targets large-class pseudo-label collapse in a source-free setting

**Table 2:** Main notation, accessible information, and task assumptions.

Symbol/Assumption	Meaning	Accessible During Adaptation
$D^{s}$	Labeled source samples from the first three dates	No
$f_{s}=g_{s}\circ h_{s}$	Trained source model; classifier $g_{s}$ and feature extractor $h_{s}$	Yes
$D^{\mathrm{ta}}$	Unlabeled Day 4 adaptation set, 3500 samples	Yes (signals only)
$D^{\mathrm{te}}$	Independent Day 4 test set, 3500 samples	No; used for evaluation after adaptation
*C* = 140	Number of closed-set transmitter classes	Yes
Target class prior	25 adaptation samples per class in the protocol; approximately uniform	Used as the balanced-marginal assumption
Target labels	True transmitter identities in $D^{\mathrm{ta}}$	No; physically removed from the file

**Table 3:** WiSig–ManyTx subset and cross-day evaluation protocol.

Item	Setting
Dataset	WiSig–ManyTx compact subset
Identification target	140 WiFi transmitters (closed-set classification)
Receiver	Fixed Rx 18-2; selected solely by data completeness
Signal version	Equalized ID signals
Input sample	256 × 2 (I/Q), float32
Source domain	1, 8, and 15 March 2021; 21,000 samples in total
Source train/validation	18,900/2100; 9:1 stratification by device × date
Target adaptation set	23 March 2021; 25 per class, 3500 total; no labels field
Independent target test set	23 March 2021; another 25 per class, 3500 total
Random seeds	42, 123, and 2026; fixed target split

**Table 4:** Source-domain ResNet1D architecture.

Stage	Configuration	Output Channels/Dimension
Input	I/Q sequence, transposed to channel-first format	2 × 256
Stem	Conv1D (*k* = 11, *s* = 2) + BN + ReLU	64
Stage 1	ResidualBlock (kernel sizes 7 and 5) × 2	64
Stage 2	ResidualBlock (64 to 128, stride = 2) + ResidualBlock	128
Stage 3	ResidualBlock (128 to 256, stride = 2) + ResidualBlock	256
Pooling	AdaptiveAvgPool1D(1)	256
Classification	Dropout(0.3) + Linear	140
Total parameters	Trainable parameters	1,860,492

**Table 5:** Key hyperparameters for source training and SF-CBPA adaptation.

Hyperparameter	Source Training	SF-CBPA Adaptation
Maximum epochs	30 (patience = 8)	10 (fixed)
Batch size	256	256
Optimizer	AdamW	AdamW
Initial learning rate	1 × 10^−3^	5 × 10^−5^
Weight decay	1 × 10^−4^	1 × 10^−4^
Learning-rate schedule	CosineAnnealing	Fixed
Label smoothing	0.1	—
Adaptation-specific parameters	—	m=0.99, ρ=0.8 $\lambda_{\mathrm{IM}}=0.1$
Gradient clipping	—	5
Sinkhorn entropy parameter *ε*	—	0.50
Sinkhorn normalization	—	50 fixed iterations
Sinkhorn stopping criterion	—	None

**Table 6:** Main results over three random seeds (mean ± sample standard deviation).

Method	Accuracy (%)	Precision	Recall	Macro-F1
Source-only	57.93 ± 0.58	0.5733 ± 0.0123	0.5793 ± 0.0058	0.5508 ± 0.0075
AdaBN	58.38 ± 0.24	0.5650 ± 0.0130	0.5838 ± 0.0024	0.5537 ± 0.0064
TENT-style	58.58 ± 0.43	0.5693 ± 0.0146	0.5858 ± 0.0043	0.5564 ± 0.0085
SHOT	61.72 ± 1.95	0.6181 ± 0.0313	0.6172 ± 0.0195	0.5963 ± 0.0285
SF-CBPA (ours)	63.80 ± 0.90	0.6462 ± 0.0141	0.6380 ± 0.0090	0.6330 ± 0.0121

**Table 7:** Exact McNemar tests comparing SF-CBPA with the three original baselines for each random seed.

Seed	Baseline	Accuracy Gain (pp)	*b*	*c*	*p*-Value
42	Source-only	+7.09	42	290	9.78 × 10^−47^
42	AdaBN	+6.17	38	254	2.20 × 10^−40^
42	TENT-style	+5.69	42	241	4.25 × 10^−35^
123	Source-only	+5.40	45	234	5.62 × 10^−32^
123	AdaBN	+4.83	40	209	8.58 × 10^−29^
123	TENT-style	+4.71	40	205	6.81 × 10^−28^
2026	Source-only	+5.11	56	235	3.05 × 10^−27^
2026	AdaBN	+5.26	42	226	1.19 × 10^−31^
2026	TENT-style	+5.26	44	228	4.09 × 10^−31^

**Table 8:** Nested block-level ablation results for SF-CBPA (seed 123). A check mark (√) indicates that the component is used, and an em dash (—) indicates that it is not used. “Entropy” denotes conditional-entropy minimization only, rather than the full IM objective.

Method	AdaBN	PL	CBPL	IM	Accuracy (%)	Macro-F1
Source-only	—	—	—	—	57.54	0.5422
AdaBN	√	—	—	—	58.11	0.5494
TENT-style	—	—	—	Entropy	58.23	0.5509
AdaBN + PL	√	√	—	—	59.91	0.5690
AdaBN + PL + IM	√	√	—	√	60.03	0.5709
AdaBN + CBPL	√	—	√	—	62.91	0.6240
SF-CBPA	√	—	√	√	62.94	0.6242

**Table 9:** One-factor component ablation with all other settings fixed (seed 123).

Variant	EMA	Sinkhorn	Within-Class Selection	IM	Accuracy (%)	Macro-F1
Full SF-CBPA	Yes	Yes	Yes	Yes	62.94	0.6242
Without EMA	No	Yes	Yes	Yes	63.43	0.6351
Without Sinkhorn	Yes	No	Yes	Yes	60.49	0.5838
Without within-class selection	Yes	Yes	No	Yes	62.71	0.6173
Without IM	Yes	Yes	Yes	No	62.91	0.6240

**Table 10:** Sensitivity to ρ and $\lambda_{\mathrm{IM}}$ with other settings fixed (seed 123).

Parameter	Value	Accuracy (%)	Macro-F1	Delta vs. Default (pp)
ρ	0.6	63.46	0.6310	+0.51
ρ	0.8 (default)	62.94	0.6242	0.00
ρ	1.0	62.71	0.6173	−0.23
$\lambda_{\mathrm{IM}}$	0.0	62.91	0.6240	−0.03
$\lambda_{\mathrm{IM}}$	0.1 (default)	62.94	0.6242	0.00
$\lambda_{\mathrm{IM}}$	0.2	63.14	0.6266	+0.20

**Table 11:** Sensitivity to the number of unlabeled target adaptation samples (seed 123).

*K* per Class	Total Unlabeled Samples	Method	Accuracy (%)	Macro-F1
0	0	Source-only	57.54	0.5422
5	700	SF-CBPA	58.63	0.5577
10	1400	SF-CBPA	60.40	0.5841
15	2100	SF-CBPA	61.71	0.6039
25	3500	SF-CBPA	62.94	0.6242

**Table 12:** Model complexity and implementation efficiency.

Item	Value	Description
Total/inference parameters	1,860,492	ResNet1D; unchanged before and after adaptation
SF-CBPA adaptable parameters	1,824,512	Classifier frozen; feature extractor updated
TENT-style adaptable parameters	4480	BN affine parameters only
Model checkpoint	7.14 MB	float32 state_dict
Batched throughput	20,223 samples/s	RTX 2060, batch = 256
Amortized time	0.049 ms/sample	Batched forward pass; not equivalent to per-sample end-to-end latency

**Table 13:** Research questions, direct evidence, and supported conclusions.

Research Question	Direct Evidence	Supported Conclusion	Unsupported Extrapolation
RQ1: Cross-day degradation	Source-only 57.93 ± 0.58%	Substantial domain shift is present on Day 4	All receivers/protocols degrade by the same magnitude
RQ2: Unlabeled adaptation	SF-CBPA 63.80 ± 0.90%; SHOT 61.72 ± 1.95%; higher for all matched seeds	Outperforms the evaluated source-free/test-time baselines under this protocol	Achieves SOTA among all source-free methods
RQ3: Component contribution	Count SD 11.75 → 3.42 after Sinkhorn; without Sinkhorn: −2.45 pp	Sinkhorn is the clearest tested component; EMA gain is not established	Every component is independently effective under arbitrary combinations
RQ4: Data and cost	Monotonic gain for *K* = 5 → 25; 7.14 MB model	Batch edge updating is feasible	Per-sample online latency or open-set performance has been validated

**Table 14:** Principal threats to validity, current controls, and future validation directions.

Threat Type	Specific Risk	Current Control	Further Validation Required
Internal validity	Target labels or test data influence adaptation	The labels field is removed at file level; the test set is read only after adaptation	Third-party blind testing and preregistered hyperparameters
Construct validity	Accuracy masks performance on difficult classes	Macro-F1, precision, recall, and the confusion matrix are all reported	Device-level confidence intervals, calibration, and rejection metrics
Conclusion validity	A single random run exaggerates the gain	Three seeds, McNemar tests, and paired bootstrap analysis	More training seeds and independent repeated collections
External validity	One dataset/receiver and a balanced prior	Explicit restriction to the fixed-Rx ManyTx-140 protocol	Cross-Rx, cross-distance, unequalized, and open-set evaluation
Implementation validity	Throughput is misinterpreted as online latency	Explicitly reported as amortized throughput with batch = 256	End-to-end latency, energy consumption, and adaptation time

## Data Availability

The original contributions presented in the study are included in the article; further inquiries can be directed to the corresponding author.
